# Response of vegetation variation to climate change and human activities in semi-arid swamps

**DOI:** 10.3389/fpls.2022.990592

**Published:** 2022-09-27

**Authors:** Guangyi Deng, Jin Gao, Haibo Jiang, Dehao Li, Xue Wang, Yang Wen, Lianxi Sheng, Chunguang He

**Affiliations:** ^1^ State Environmental Protection Key Laboratory of Wetland Ecology and Vegetation Restoration, Key Laboratory for Vegetation Ecology Ministry of Education, Northeast Normal University, Changchun, China; ^2^ Key Laboratory of Environmental Materials and Pollution Control, The Education Department of Jilin Province, School of Engineering, Jilin Normal University, Siping, China

**Keywords:** temporal and spatial changes in vegetation, migration stopover, Momoge National Nature Reserve of Jilin Province, Google Earth Engine, anthropogenic activities, climate variation

## Abstract

Vegetation is a sensitive factor in marsh ecosystems, which can provide nesting sites, foraging areas, and hiding places for waterfowl and can affect their survival environment. The Jilin Momoge National Nature Reserve, which consists of large areas of marshes, is located in the semi-arid region of northeast China and is an important stopover site for the critically endangered species of the Siberian Crane (*Grus leucogeranus*). Global climate change, extreme droughts and floods, and large differences in evaporation and precipitation in this region can cause rapid vegetation succession. In recent years, increased grain production and river-lake connectivity projects carried out in this area to increase grain outputs and restore wetlands have caused significant changes in the hydrological and landscape patterns. Therefore, research on the response of variation trends in vegetation patterns to the main driving factors (climate change and human activities) is critical for the conservation of the Siberian Crane. Based on the Google Earth Engine (GEE) platform, we obtained and processed the Normalized difference vegetation index (NDVI) data of the study area during the peak summer vegetation period for each year from 1984 to 2020, estimated the annual vegetation cover using Maximum value composites (MVC) method and the image dichotomy method, calculated and analyzed the spatial and temporal trends of vegetation cover, explored the response of vegetation cover change in terms of climate change and human activities, and quantified the relative contribution of both. The results revealed that first, from the spatial and temporal changes, the average annual growth rate of regional vegetation was 0.002/a, and 71.14% of the study area was improved. The vegetation cover showed a trend of degradation and then recovery, in which the percentage of high vegetation cover area decreased from 51.22% (1984–2000) to 28.33% (2001–2005), and then recovered to 55.69% (2006–2020). Second, among climate change factors, precipitation was more correlated with the growth of vegetation in the study area than temperature, and the increase in precipitation during the growing season could promote the growth of marsh vegetation in the Momoge Reserve. Third, overall, human activities have contributed to the improvement of vegetation cover in the study area with the implementation of important ecological projects, such as the return of farmland to wetlands, the return of grazing to grass, and the connection of rivers and lakes. Fourth, climate change and human activities jointly drive vegetation change, but the contribution of human activities in both vegetation improvement and degradation areas (85.68% and 78.29%, respectively) is higher than that of climate change (14.32% and 21.71%, respectively), which is the main reason for vegetation improvement or degradation in the study area. The analysis of vegetation pattern change within an intensive time series in semi-arid regions can provide a reference and basis for studying the driving factors in regions with rapid changes in vegetation and hydrological conditions.

## 1 Introduction

Wetlands are one of the most productive ecosystems in the world and have irreplaceable ecological value for the conservation of species and the maintenance of biodiversity (Mas et al., 2021). As the core part of wetland ecosystems, wetland vegetation plays an important role in the balance of Earth’s ecosystem, climate change, the water cycle, and energy flow (e.g., Guntenspergen et al., 1989; Shen et al., 2021; Zhang et al., 2021; Shen et al., 2022c; Yan et al., 2022). At the same time, vegetation is also a sensitive factor for ecological changes and is considered to be an important part of migratory waterfowl habitat, providing food, hiding places, and breeding sites for waterfowl, directly affecting their living environment (Barchiesi et al., 2022; Wang et al., 2022c; Yuan et al., 2022). To ensure that waterfowl are provided with a suitable habitat at each stopover point along the migration corridor, it is important to effectively monitor vegetation changes and drivers of waterfowl habitat over time.

The Jilin Momoge National Nature Reserve (hereinafter referred to as Momoge Reserve), located in the western part of the Songnen Plain in China, is a long-term supply stop on the East Asia¬–Australia migratory bird route, providing *Bolboschoenus planiculmis*, an important food source for whooping cranes, before they fly to their wintering grounds (Wang et al., 2022d). The Momoge Nature Reserve is a typical climate change-sensitive area. In the context of long-term global warming and economic development, the region has suffered continuous drought and wetland development in recent years, which has continuously changed the distribution pattern of vegetation, thus affecting the distribution pattern and resting population of whooping cranes (Jiang et al., 2015a; Haverkamp et al., 2022). Although the mechanisms of vegetation dynamics are complex and multifaceted, climate change and human activities have been identified as the main drivers (Qi et al., 2022; Wu et al., 2022). Many studies have been conducted on the response of vegetation to climate change and human activities (Hu et al., 2011; Liu et al., 2015; Yuan et al., 2019; Bai et al., 2022), however, few studies have been able to quantitatively assess the long-term response of vegetation to climate change and human activities, and there is a lack of effective differentiation between the effects of climate factors and human activities on vegetation and quantification of their respective relative contributions to vegetation change (Wang et al., 2022b). Therefore, studying the mechanisms of climate change and human activities on the dynamics of vegetation change in waterfowl habitats has become a key issue for the restoration and conservation of food resources in whooping crane resting habitats.

To elucidate the vegetation changes in whooping crane stopover sites and their response to climate change and human activities, this study was conducted in the western half of the Momoge Reserve to investigate the three following aspects: (1) how vegetation patterns have changed dynamically over the past 37 years, (2) the relationship between vegetation dynamics and climate change at spatial and temporal scales, and (3) the relative contribution of climate change and human activities to vegetation dynamics and provision of theoretical guidance for ecological management and sustainable development of wetlands in the Momoge Reserve.

## 2 Materials and methods

### 2.1 Study area

**Figure 1:**
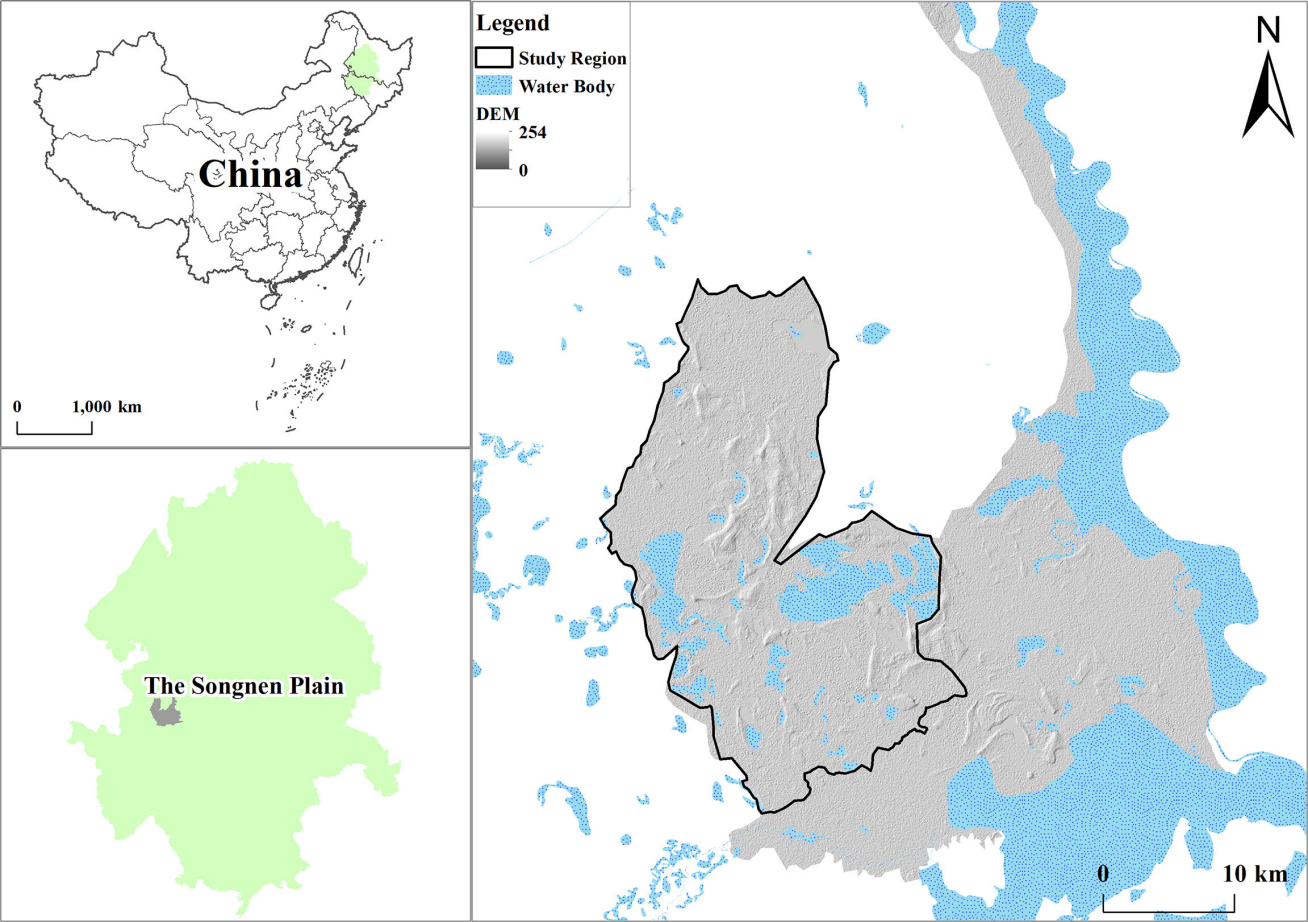
Location of study area.

The study area was located within the Momoge Reserve in northeastern China, a region where the maximum number of whooping cranes at rest represents more than 95% of the world’s population (**Figure 1**). The study area is located in the western semi-arid region of the Songnen Plain, China, and is an important stopover area along the East Asia–Australia migratory route for migratory birds (Tian et al., 2019). It was included in the list of internationally important wetlands in 2013. The region has a temperate continental monsoon climate with an annual average temperature of 4.2°C (Wang et al., 2020), annual evaporation of 1200–1900 mm, and annual average precipitation of 378.14 mm, with an extremely uneven distribution of precipitation during the year, mainly concentrated in June–August, accounting for 79% of the total annual precipitation. The Tao’er and Erlongtao Rivers flow through the area. The plant and animal resources in the reserve are rich, with more than 600 species of seed plants, including herbaceous species such as reeds, small-leafed adults, moss grasses, and fragrant cattails, which have a high population density and biomass, are the dominant species in the reserve, and are distributed in slightly different areas (Zhao et al., 2022).

### 2.2 Data source and pre-processing

The NDVI data were obtained from GEE platform, spanning 1984–2020, with a spatial resolution of 30 m. The downloaded data were pre-processed using software for stitching, projection, and format conversion. Maximum value composites (MVC) method was used to obtain the monthly NDVI data, which reduced the effects of clouds, atmosphere, and solar altitude angle (Holben, 1986), which were averaged to obtain the growing season NDVI data. And then the monthly NDVI data were extracted by the mean method from April to October each year to obtain the year-by-year growing season NDVI data.

The meteorological data were obtained from the China Meteorological Data Network(http://cdc.cma.gov.cn/), and the month-by-month hydrological and temperature data from 1984 to 2020 were extracted from the nearest meteorological stations in Baicheng City. The meteorological data were projected and resampled by ArcGIS 10.6 software to obtain the meteorological factor raster data with the same resolution as NDVI data.

Landuse/landcover (LULC) data were obtained from the Resource and Environment Science and Data Centre of the Chinese Academy of Sciences(http://www.resdc.cn), and were divided into seven phases of images (1980, 1990, 2000, 2005, 2010, 2015, and 2020) with a spatial resolution of 30 m. The land use types were grassland, swampy wetland, water surface, paddy field, dry field, artificial surface, sandy land, saline land, and woodland

### 2.3 Methods

#### 2.3.1 Fractional vegetation cover

A like-element dichotomous model was used to estimate the fractional vegetation cover (FVC) in the study area (Gong et al., 2017) to explore the spatial and temporal variation characteristics of vegetation cover using the following equation:


(1) $$FVC=\frac{\left(NDVI-NDVI_{soil}\right)}{\left(NDVI_{veg}-NDV_{soil}\right)}$$

where FVC is the vegetation cover, *NDVI*_*soil*_ is the NDVI value of bare land, and *NDVI*_*veg*_ is the NDVI value of high cover. In this study, the normalized vegetation index with a cumulative frequency of 5% was selected as *NDVI*_*soil*_ , and the normalized vegetation index with a cumulative frequency of 95% was selected as *NDVI*_*veg*_ . Based on the grading standards of vegetation cover in the northeastern black soil area in the Soil Erosion Classification and Grading Standards issued by the Ministry of Water Resources of China in 2008, and combined with the characteristics of the local ecological environment (Jiang et al., 2017), the proportional standards of vegetation cover were developed and classified into five categories: low coverage, low to medium coverage, medium coverage, medium to high coverage, and high coverage (**Table 1**).

**Table 1:** The classification standard of FVC.

Grade	FVC	Classification features
Low	<10%	Basically no vegetation
Low to medium	10%-30%	Sparse vegetation
Medium	30%-45%	Better vegetation
Medium to high	45%-60%	Well-vegetated
High	≥60%	Lush vegetation

#### 2.3.2 Trend analysis

The Theil–Sen Median trend analysis estimation was used to explore the trend of NDVI of the vegetation in the study area (Jiang et al., 2015b; Jiang et al., 2022) to reveal its change characteristics on the time scale. The formula used is as follows:


(2) $$\theta_{NDVI}=Median\left(\frac{NDVI_{j}-NDVI_{i}}{j-i}\right)$$

Where *i* and *j* represent years, *i* ≤ *j* , and takes values between 1984 and 2020. *NDVI*_*i*_ and *NDVI*_*j*_ represent NDVI in years i and j, respectively. *θ*_*NDVI*_  represents the slope of the change in NDVI of vegetation during the study period. When *θ*_*NDVI*_> 0 , the NDVI of vegetation during the study period was considered to be increasing and the vegetation cover was improving. When *θ*_*NDVI*_> 0 , the vegetation NDVI remained unchanged and the vegetation cover was more stable; when *θ*_*NDVI*_> 0 , the vegetation NDVI decreased, and the vegetation cover was degraded during the study period.

The Mann–Kendall significance test was used to determine whether the slope of change in vegetation NDVI over the study period was significant and the degree of significance (Mann, 1945; Kendall, 1948). The results of *θ*_*NDVI*_  and Mann–Kendall significance test were combined to obtain six scenarios of vegetation NDVI: when *θ*_*NDVI*_> 0 and passed *α*=0.05 significance test, vegetation NDVI showed a significant increasing trend; when *α*=0.05 significance test was not passed, vegetation NDVI showed a non-significant increasing trend; similarly, vegetation NDVI showed a decreasing trend. Two scenarios with decreasing trends in NDVI were obtained.

#### 2.3.3 Partial correlation analysis

The partial correlation between vegetation cover and each climate factor was analyzed using image-based spatial analysis (Yuan et al., 2019). The simple correlation coefficient was calculated first, and then the partial correlation coefficient was obtained. The correlation coefficients of NDVI, temperature, and rainfall were calculated as follows:


(3) $$r_{xy}=\;\frac{\sum_{1=1}^{n}(x_{1}-\bar{x})(y_{1}-\bar{y})}{\sqrt{\sum_{1=1}^{n}{\left(x_{1}-\bar{x}\right)}^{2}{\left(y_{1}-\bar{y}\right)}^{2}}}$$

where r_xy_ is the correlation coefficient between NDVI and climate factor, *x_i,_ y_i_* are the values of climate factor and vegetation NDVI in year *i* respectively, *x_i_, yi* are the mean values of each variable, *i* is the year number, and *n* is the monitoring year.

Partial correlation analysis was used to analyze the degree of correlation between two variables, without considering the effect of the third variable. The partial correlation coefficient is calculated based on a linear correlation using the following formula:


(4) $$r_{xy,z}=\;\frac{r_{xy}-r_{xz}r_{yz}}{\sqrt{\left(1-r_{xy}^{2})(1-r_{yz}^{2}\right)}}$$

where *r_xy,z_* denotes the partial correlation coefficient of *x* and *y* variables after the fixed variable *z*; *r_xy_*, *r_xz_*, *r_yz_* are the correlation coefficients between the respective variables, and the partial correlation coefficients take values in the range of –1 to 1.

The test of correlation coefficient is often done by T-test and according to the test results we can classify the bias correlation into 6 levels: highly significant positive correlation (*r_xy,z_* > 0,*p<* 0.01), highly significant negative correlation (*r_xy,z_* < 0,p< 0.01), significant positive correlation (*r_xy,z_* > 0,0.01 ≤ p< 0.05), significant negative correlation(*r_xy,z_* < 0,0.01 ≤ p< 0.05), no significant positive correlation (*r_xy,z_* > 0,p ≥ 0.05), no significant negative correlation (*r_xy,z_* < 0,p ≥ 0.05).

#### 2.3.4 Relative role analysis

Residual analysis has been used to discern the effects of human activities on vegetation changes (Wessels et al., 2007). NDVI predictions were obtained by fitting precipitation and air temperature data to obtain a time series of NDVI for the vegetation growing season with only climatic effects, thus obtaining the residual value, which is the effect of human activity on NDVI for the vegetation growing season, using the following equation:


(5) $$NDVI_{Pre}=\;a\times P\times b\times T\;+C$$

(6) $$NDVI_{Rre}=\;NDVI_{Real}-NDVI_{Pre}$$

where *NDVI_Pre_* denotes the predicted value of NDVI, *NDVI_Real_* denotes the real value of NDVI; *α* and *b* denote the regression coefficients of NDVI on precipitation and temperature, respectively; *C* denotes the regression constant term; *P* denotes precipitation; and *T* denotes temperature. An *NDVI_Res_* > 0 indicates a positive effect of human activities, whereas an *NDVI_Res_* = 0 indicates a negative effect of human activities. An *NDVI_Res_* = 0 indicates a relatively weak effect of human activities.

The test of residual values is often done by T-test and according to the test results we can classify residual values into 4 levels: significant positive correlation (*NDVI_Res_* > 0,p<0.05), significant negative correlation (*NDVI_Res_* < 0,p<0.0)5, no significant positive correlation (*NDVI_Res_* > 0,p ≥ 0.05), no significant negative correlation. (*NDVI_Res_* < 0,p ≥ 0.05).

#### 2.3.5 Driving factor determination

Using the simulation assessment method (Cheng et al., 2021), the trends of *NDVI_Real_*, *NDVI_Pre_*, and *NDVI_Res_* ( *θ*_*NDVI*_*Real*__ , *θ*_*NDVI*_*Pre*__ , *θ*_*NDVI*_*Res*__ ) were calculated using residual analysis, and the spatial distribution of the drivers was calculated using ArcGIS 10.6 (**Table 2**).

**Table 2:** Determination criteria of driving factors.

*θ*_*NDVI*_*Real*__	Driving Factors	*θ*_*NDVI*_*CC*__	*θ*_*NDVI*_*HA*__	Contribution of drivers (%)	
**CC**	**HA**
**>0**	CC&HA	>0	>0	$\frac{\theta_{NDVI_{CC}}}{\theta_{NDVI_{Real}}}$	$\frac{\theta_{NDVI_{HA}}}{\theta_{NDVI_{Real}}}$
	CC	>0	<0	100	0
	HA	<0	>0	0	100
**<0**	CC&HA	<0	<0	$\frac{\theta_{NDVI_{CC}}}{\theta_{NDVI_{Real}}}$	$\frac{\theta_{NDVI_{HA}}}{\theta_{NDVI_{Real}}}$
	CC	<0	>0	100	0
	HA	>0	<0	0	100

HA, human activity; CC, natural factor; θ_NDVI_CC__ refers to the trend of climate change impact ( θNDVI_Real_), θ_NDVI_HA__ refers to the trend of human activity impact ( θNDVI_Pre_), and θNDVI_Real_ refers to the trend of actual NDVI change.

#### 2.3.6 Relative action method

Relative contribution analysis was used to quantify the relative contributions of climate change and human activity to vegetation changes (Sun et al., 2020). Residual analysis was used to establish the evaluation method of the relative contribution of climate change and human activities to the vegetation change process under different possible scenarios (**Table 3**). When *θ*_*NDVI*_*Real*__ > 0 , it indicates that vegetation tends to improve, and vice versa; when *θ*_*NDVI*_*Pre*__ > 0 , it indicates that climate change promotes vegetation growth, and vice versa; and when *θ*_*NDVI*_*Res*__ > 0 , it indicates that human activities promote vegetation growth, and vice versa.

**Table 3:** Methods for evaluating the relative roles of climate change and human activities in the process of vegetation change under different scenarios.

Zoning	Scenario	*θ*_*NDVI*_*Pre*__	*θ*_*NDVI*_*Res*__	The relative role of climate change × 10000	The relative role of human activities × 10000	Description
Vegetation improvement	1	>0	>0	*θ*_*NDVI*_*Real*__ $\frac{|\Delta NDVI_{Pre}}{|\Delta NDVI_{Pre}+\;\Delta NDVI_{Res}}$	$\frac{|\Delta NDVI_{Res}}{|\Delta NDVI_{Pre}+\;\Delta NDVI_{Res}}$	Common driven
	2	>0	<0	100%	0%	CC driven
	3	<0	>0	0%	100%	HA driven
Vegetation degradation	4	<0	<0	$\frac{|\Delta NDVI_{Pre}}{|\Delta NDVI_{Pre}+\;\Delta NDVI_{Res}}$	$\frac{|\Delta NDVI_{Res}}{|\Delta NDVI_{Pre}+\;\Delta NDVI_{Res}}$	Common driven
	5	<0	>0	100%	0%	CC driven
	6	>0	<0	0%	100%	HA driven

ΔNDVI_Pre_ is the difference between NDVI_Pre_ and in year t+1 under the influence of climate change; is the difference between. NDVI_Res_ and NDVI_Res_ in year t+1 under the influence of human activities.

#### 2.3.7 LULC transfer matrix analysis

The spatial transformation of land use types was analyzed for the study area in 1980, 1990, 2000, 2005, 2010, 2015, and 2020 using a transfer matrix (Yao et al., 2021). The wetland transfer matrix was visualized using a Sankey diagram with the following equation.


(7) $$S_{ij}=\left(\begin{array}{l} S_{11}\;S_{12}\;\ldots \;S_{1n}\\ S_{21}\;S_{22}\;\ldots \;S_{2n}\\ \;\ldots \;\ldots \;\ldots \;\ldots \\ S_{n1\;}\;S_{n2}\;\ldots \;S_{nn} \end{array}\right)\;$$

Where *S* is the area; *n* is the number of landscape types; and *i*, *j* in *S_ij_* denote the landscape types at the beginning and end of the study period, respectively.

## 3 Results

### 3.1 FVC change characteristics

#### 3.1.1 Temporal and spatial analysis of FVC

**Figure 2:**
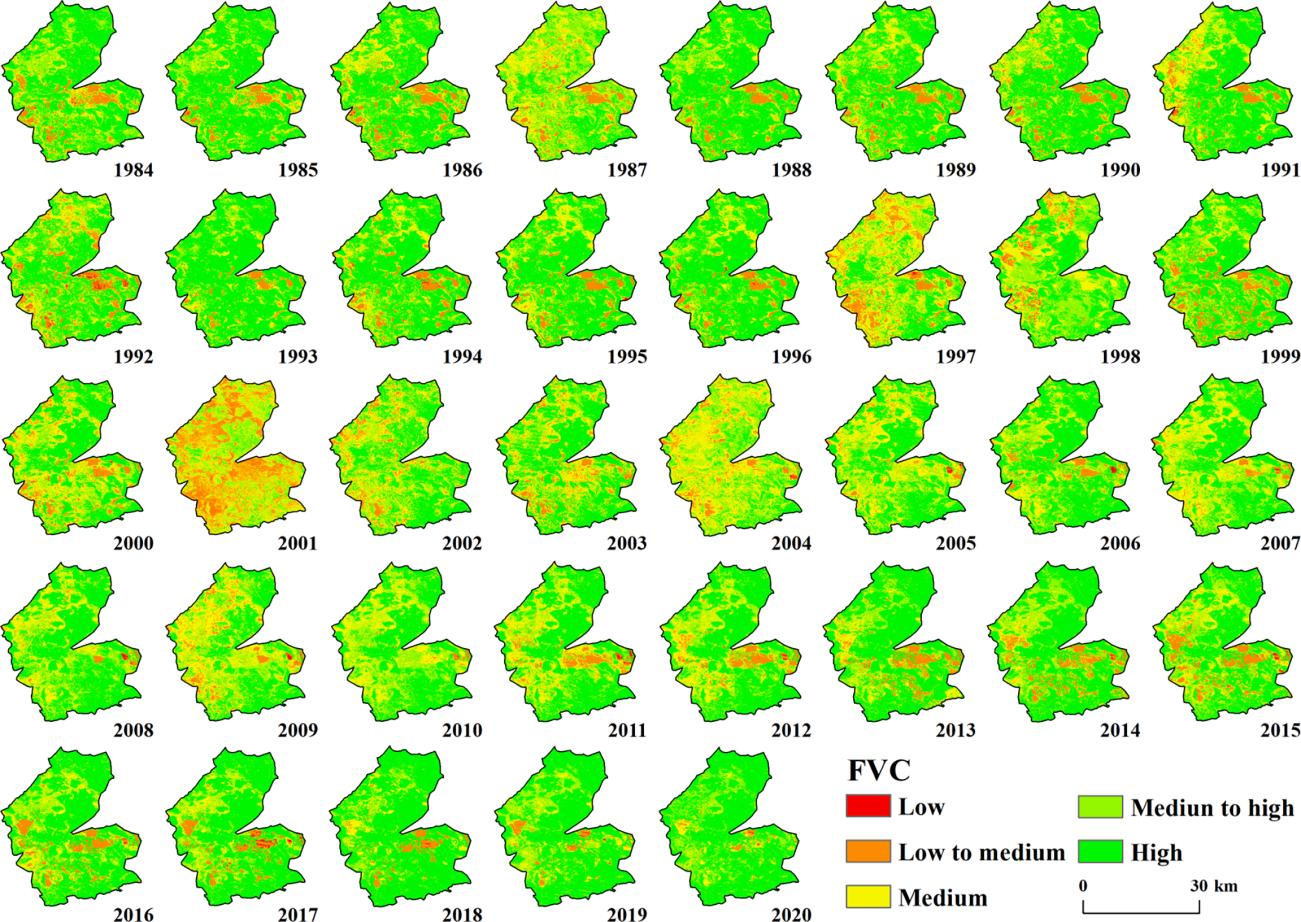
Spatial distribution characteristics of FVC in the study area from 1984–2020.

The overall vegetation cover in the study area from 1984 to 2020 was good, and the area was dominated by medium to high vegetation cover. Additionally, there was heterogeneity in the spatial cover of vegetation between different years (**Figure 2**). The first period (1984–2000) had a wide area of vegetation cover and more areas of high and medium vegetation cover, which were evenly distributed in the study area. Medium vegetation cover was distributed in the northern part of the study area, while there were less low and medium vegetation cover areas, which were mainly scattered in the southern part of the study area and the western part, and the low vegetation cover areas were scattered. In the second period (2001–2005), there was a decrease in vegetation cover in the study area, low vegetation cover, medium-low vegetation cover, and medium vegetation cover gradually expanded, medium vegetation cover areas were evenly distributed in the study area, medium-low vegetation cover spread to the northern part of the study area, and low vegetation cover was mainly in the west, especially in 2001; the medium-low vegetation cover area was the highest. The third period covered the period from 2006–2020, and the high vegetation cover and medium vegetation cover in this time period again increased significantly each year. The vegetation in the northern area grew and returned to the high vegetation area and medium-high vegetation cover area. Compared with the first period, the medium vegetation cover and medium-low vegetation cover area were less, and the medium-low vegetation cover was mainly located in the southern part of the study area.

**Figure 3:**
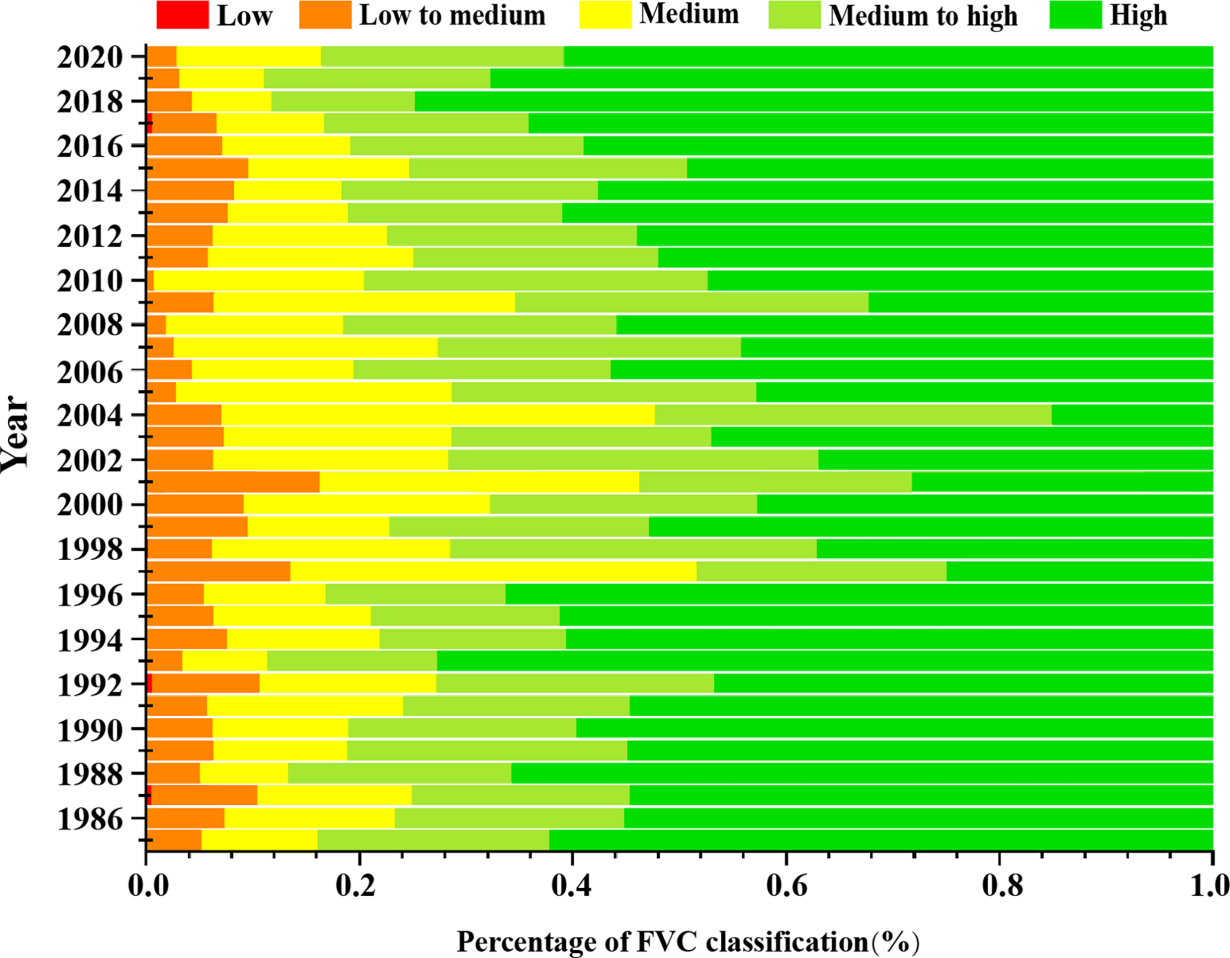
The proportion of FVC of different grades in the study area from 1984–2020.

The differences in the different classes of vegetation cover in the different years were more obvious (**Figure 3**). Overall, high vegetation cover was the most distributed, with an average of 49.94%, followed by medium-high vegetation cover with an average of 22.97%, medium value cover with an average of 16.99%, medium-low vegetation with an average of 9.81%, and low vegetation cover with an average of 0.28%. The study period is divided into 3 stages. The first period spanned 1984–2000. During this period, the average percentage of high vegetation cover area was 51.22%, the average percentage of higher vegetation cover was 21.21%, average percentage of medium vegetation cover was 15.02%, average percentage of medium and low vegetation cover area was 12.09%, and average percentage of low vegetation cover area was 0.47%. It can be seen that the percentage of high vegetation cover is the highest. In the second period, from 2001 to 2005, the average value of high vegetation cover area was 28.33%, average value of higher vegetation cover area was 26.23%, average value of medium vegetation cover area was 29.19%, average value of medium and low vegetation cover area was 16.18%, and average value of low vegetation cover area was 0.05%. Thus, it was found that the high vegetation cover area was still the highest, but with a decreasing trend; the mean value is 16.7% less than in the first stage, but the distribution of medium vegetation and medium-high vegetation increased significantly and the proportion of medium-low vegetation cover decreased. The third period was from 2006 to 2020; in this period, the mean value of the high vegetation cover area was 55.69%, the mean value of higher vegetation cover was 23.89%, proportion of medium vegetation cover average value was 15.16%, the average value of the medium-low vegetation cover area was 5.12%, and the average value of the low vegetation cover area was 0.15%. The distribution of the high vegetation area increased significantly, and the percentage of medium-low vegetation cover continued to show a decreasing trend. In summary, the change in low vegetation cover was small in the three stages, medium-low vegetation cover showed a decreasing trend, medium vegetation cover and medium-high vegetation cover showed roughly the same trend, that is, increasing and then decreasing, while high vegetation cover showed a decreasing and then increasing trend.

#### 3.1.2 NDVI trend analysis

**Figure 4:**
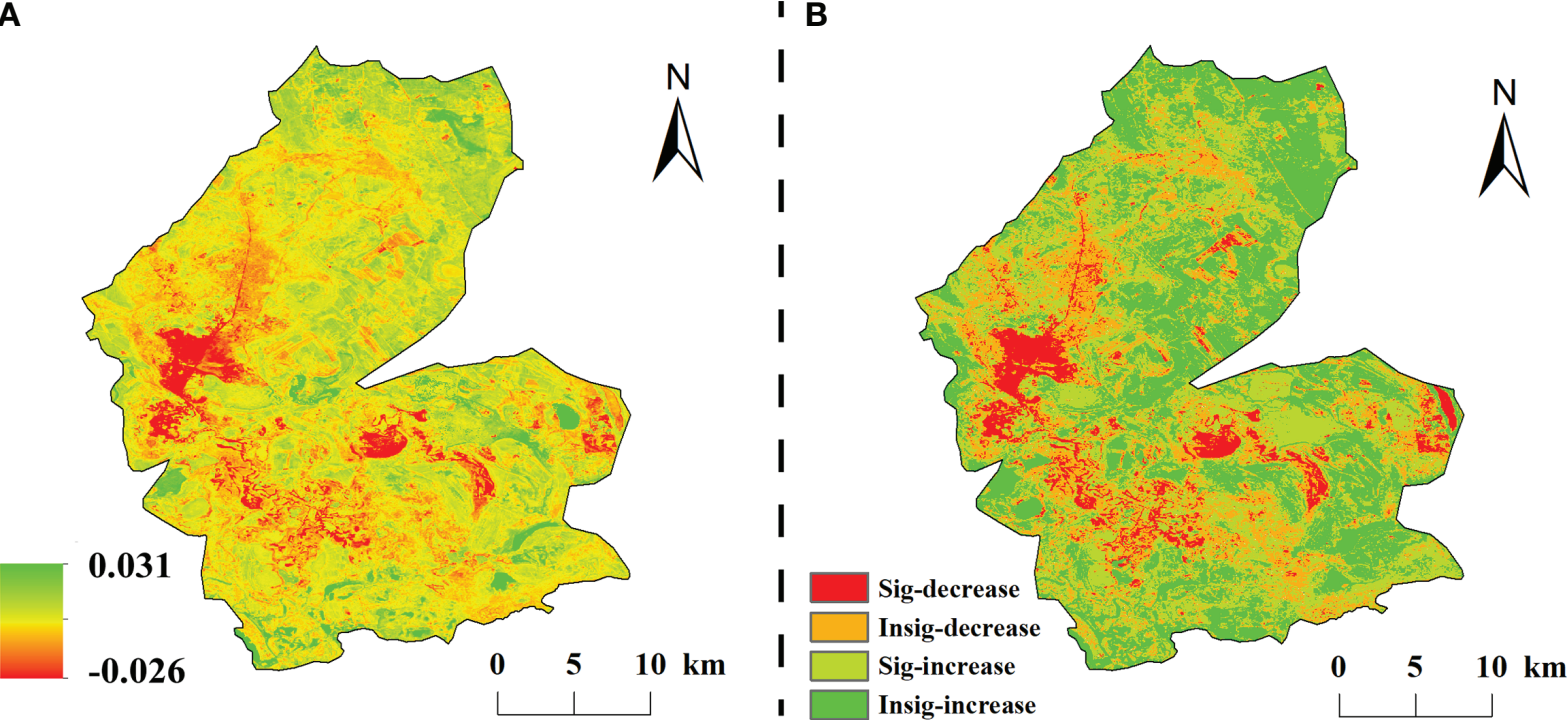
The classification of change trend in the study area from 1984 to 2020. **(A)** is the slope of interannual variation of NDVI, **(B)** is the spatial distribution of significance.

The spatial and temporal variation of vegetation NDVI in the study area from 1984 to 2020 showed obvious spatial heterogeneity (**Figure 4**). The multi-year average NDVI change rate was –0.026–0.031/a, with a mean value of 0.002/a. 71.14% of the vegetation cover in the study area was improved, among which, 37.54% of the significantly improved areas were evenly dispersed in the study area; while The degraded areas accounted for 28.86% and only 7.2% of the significantly degraded areas, which were mainly concentrated in the central part of the study area.

### 3.2 Response of vegetation change to climatic factors

#### 3.2.1 Analysis of interannual variability of climatic factors

**Figure 5:**
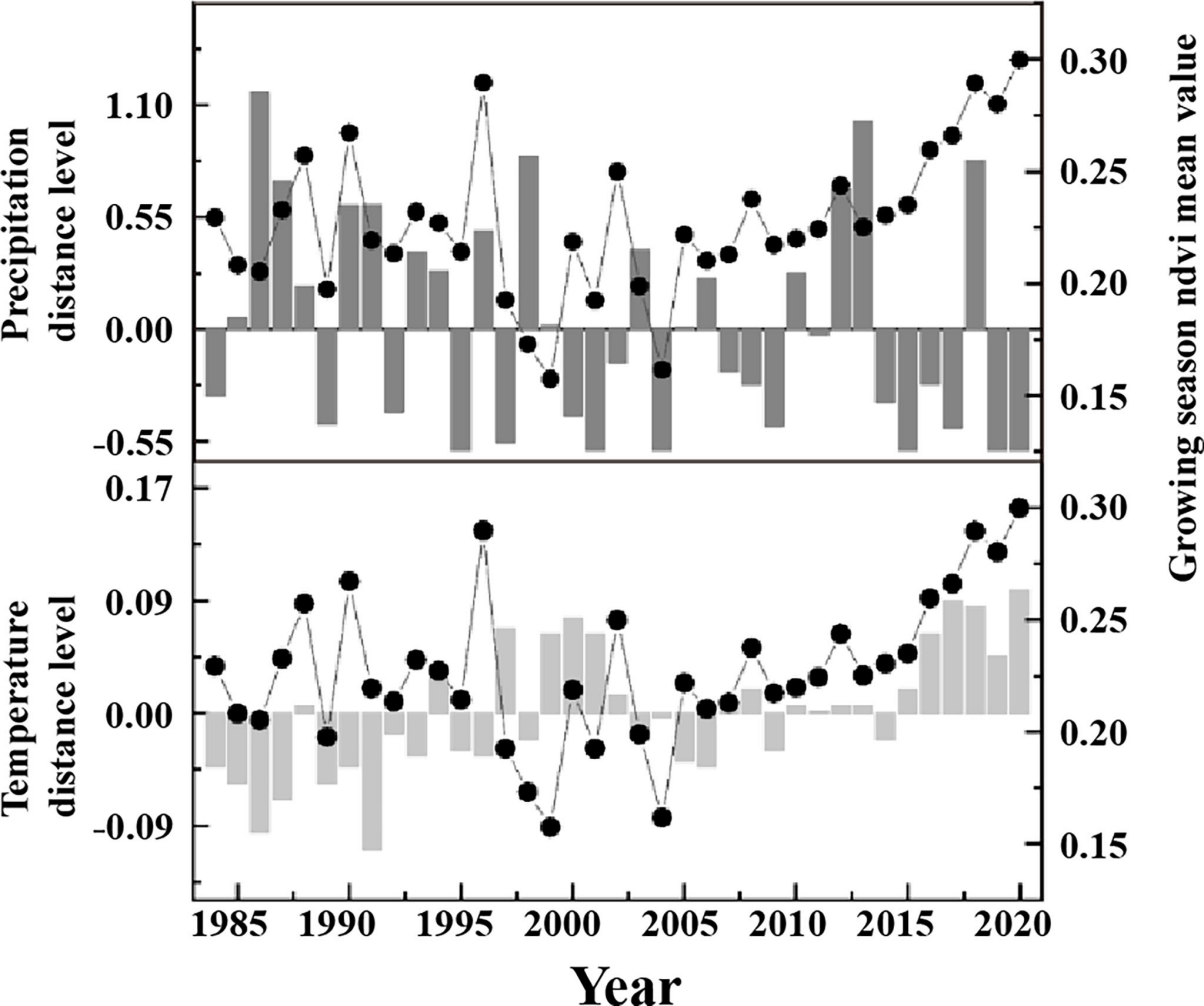
The interannual variations of NDVI, precipitation and temperature in June of 1984-2020.

The vegetation growing seasons in the study area from 1984 to 2020 were generally consistent with temperature changes during the same period (**Figure 5**). The growing season NDVI trend in the study area shifted in 2000, that is, increasing and then decreasing, and then shifted a second time in 2006, from decreasing to increasing, and the rate of increase was higher than the rate of increase before the 1990s. The growing season temperature in the study area also showed decreasing, increasing, and substantially increasing trends from to 1984–2000, 2001–2005, and 2006–2020, respectively, which were almost consistent with the fluctuations and trends of NDVI.

#### 3.2.2 Correlations of NDVI with temperature and precipitation

**Figure 6:**
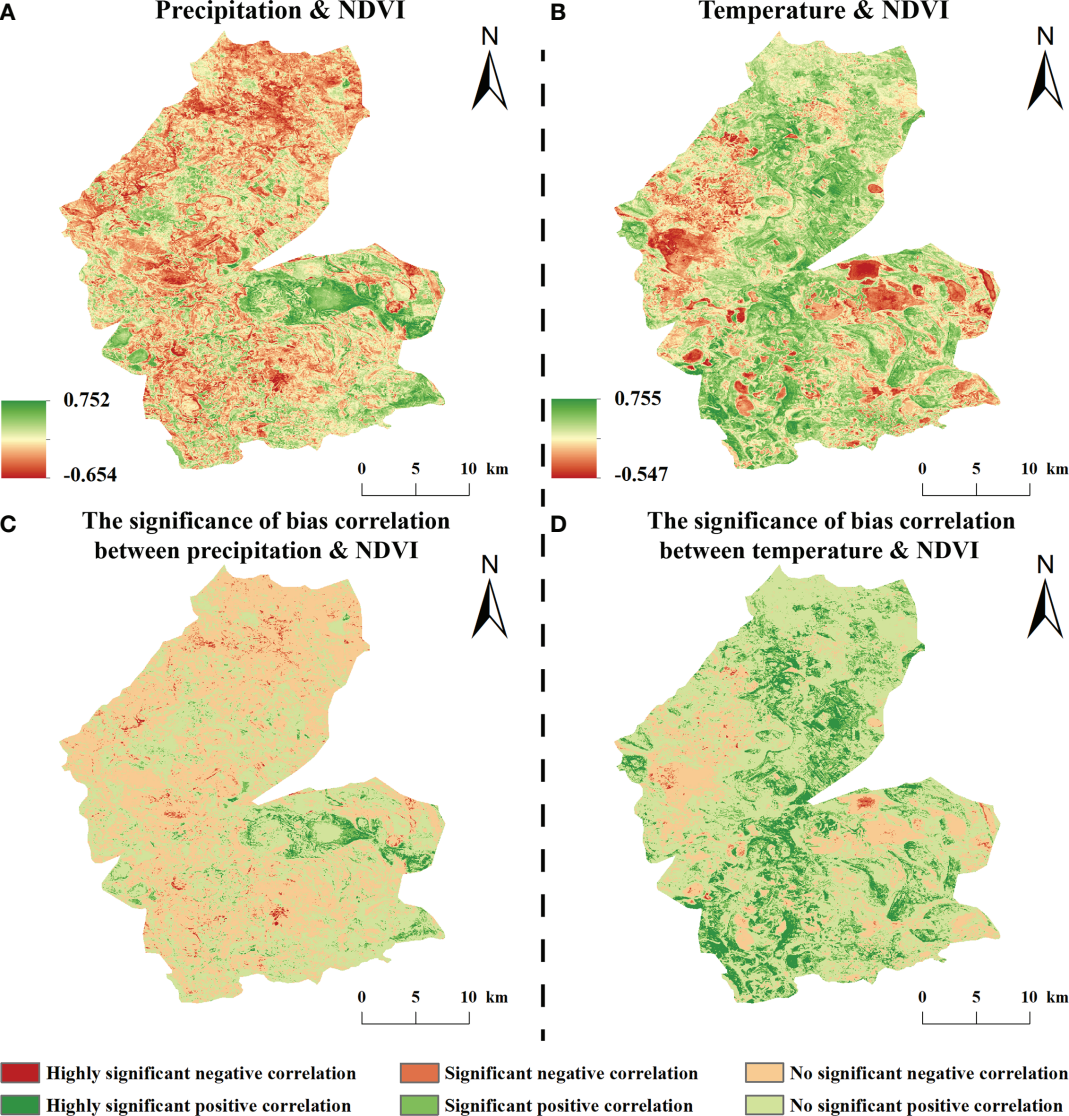
Bias correlation between NDVI and climatic factors from 1984-2020 (**A, B** are the bias off NDVI with precipitation and temperature, respectively; **C, D** are the respective significance).

The correlation coefficient between the NDVI and precipitation for the entire study area was 0.19. The correlation coefficient between NDVI and precipitation in the growing season of vegetation in the study area was mainly positive, ranging from –0.546 to 0.755, with positive and negative correlations accounting for 53.89% and 46.11% of the study area, respectively. Significant negative correlations accounted for 3.68% and were sporadically distributed within the study area. Insignificant positive and negative correlations accounted for 50.66% and 42.43%, respectively, and were evenly distributed in the study area (**Figure 6**).

The NDVI and temperature in the study area mainly showed a positive correlation during the growing season, with correlation coefficients of –0.654 to 0.752, of which the proportion of positive and negative correlation areas were 52.79% and 47.21% of the whole study area, respectively; among them, a significant positive correlation accounted for 2.14% and was scattered in the study area; a significant negative correlation accounted for 4.78% and was mainly distributed in the central part of the study area; an insignificant positive correlation accounted for 50.66%, mainly in the western and eastern regions of the study area, and 42.43% of the insignificant negative correlations were scattered in the study area (**Figure 6**).

### 3.3 Response of vegetation change to human activities

#### 3.3.1 Residual analysis

**Figure 7:**
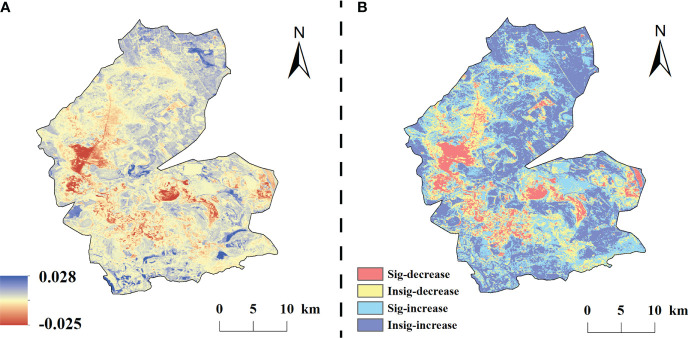
Trends in NDVI residuals under the influence of human change from 1984–2020 (**A** is the residual trend, **B** is the residual significance).

On a time scale, the mean values of NDVI residuals of vegetation in the study area were mainly negative in the early period and positive beginning in 2010. The residuals showed an overall increasing trend at a rate of 0.0021a^-1^. Spatially (**Figure 7**), the NDVI residual values in the study area varied from –0.0.028 to 0.025 a^-1^, and the areas with positive residuals accounted for 71.50% of the total area, of which 34.42% of the total area was significantly increased and concentrated in the northern and eastern parts of the study area.

#### 3.3.2 LULC impacts on vegetation pattern changes

**Figure 8:**
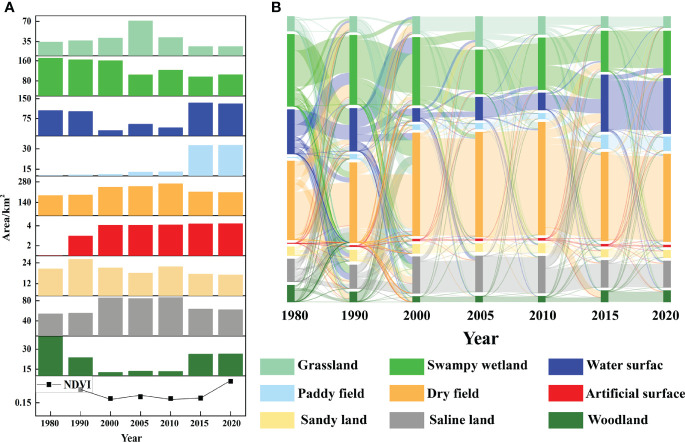
The change of land use types and NDVI values in the study area (**A** is the trends in land use area and NDVI values, **B** is the sankey diagram of landscape transfer matrix).

The correlation between land use change and vegetation change in the study area from 1980 to 2020 was clearly characterized (**Figure 8A**). The area changes of herbaceous wetlands, water bodies and woodlands were consistent with the trend of NDVI change, decreasing first and then increasing, while the area changes of grassland, dryland and saline land were opposite to the trend of NDVI change. In addition, land use types with different NDVI transformed into each other to different degrees during this period (**Figure 8B**). From 1980 to 1990, a total of 95.12 km^2^ was transferred from grassland, swampy wetland, water, saline land and forest land to dryland, indicating that there was more agricultural reclamation during this period; from 1990 to 2000, the transfer from swampy wetland, water, dryland and sandy land to saline land 57.68 km^2^, indicating that a part of the land began to be saline in that period; from 2000 to 2005, the areas of marsh wetland to water surface, grassland and dryland were 32.71 km^2^, 20.08 km^2^ and 9.45 km^2^, indicating that a large area of marsh wetland was degraded in that period; from 2005 to 2010, the areas of grassland to dryland and marsh wetland were 15.33 km^2^, 13.19 km^2^; indicating that both agricultural reclamation and ecological water replenishment projects existed simultaneously in this period; from 2010 to 2015, the area shifted from marsh wetland, dryland and saline land to water bodies was 48.50 km^2^, 21.23 km^2^, 16.60 km^2^, indicating that ecological restoration efforts were stronger in this period; from 2015 to 2020, water surface to marsh wetland shifted 11.12 km^2^, indicating the existence of vegetation protection in this period.

### 3.4 Vegetation change driving mechanisms

#### 3.4.1 Spatial distribution of drivers of vegetation change

**Figure 9:**
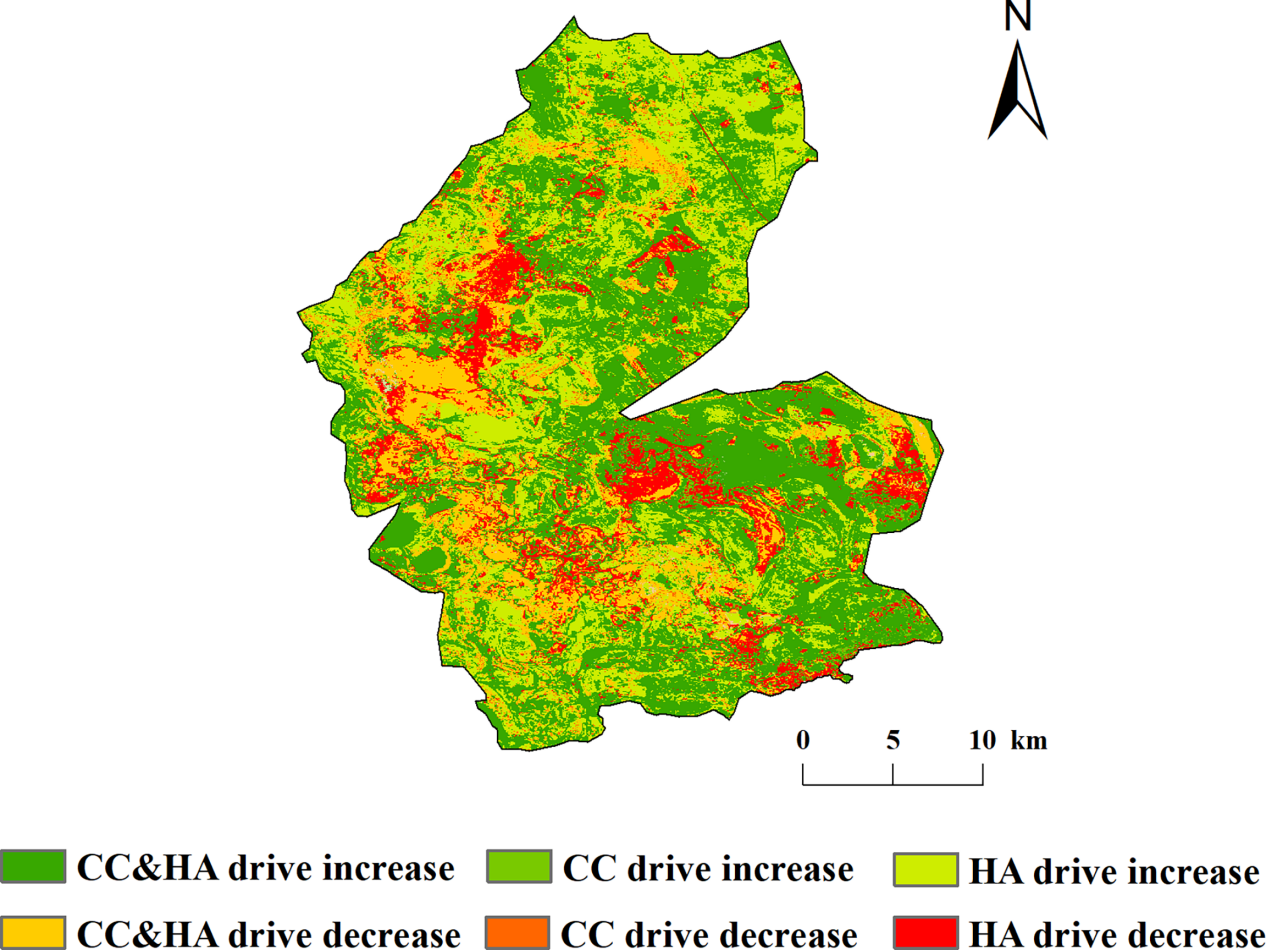
Spatial distribution of driving mechanisms of vegetation NDVI change in the study area.

The percentage of areas where the NDVI of vegetation in the study area was driven by both climate change and human activity was 43.23%, which was mainly distributed in the eastern part of the study area. The percentages of NDVI increase in vegetation driven by climate change alone and by human activity alone were 1.78% and 26.72%, respectively. By contrast, 15.12%, 1.71%, and 11.44% of the areas driven by climate change and human activities, climate change alone, and human activities alone, respectively, were distributed in the western part of the study area (**Figure 9**).

#### 3.4.2 Relative contribution of different drivers to vegetation change

**Figure 10:**
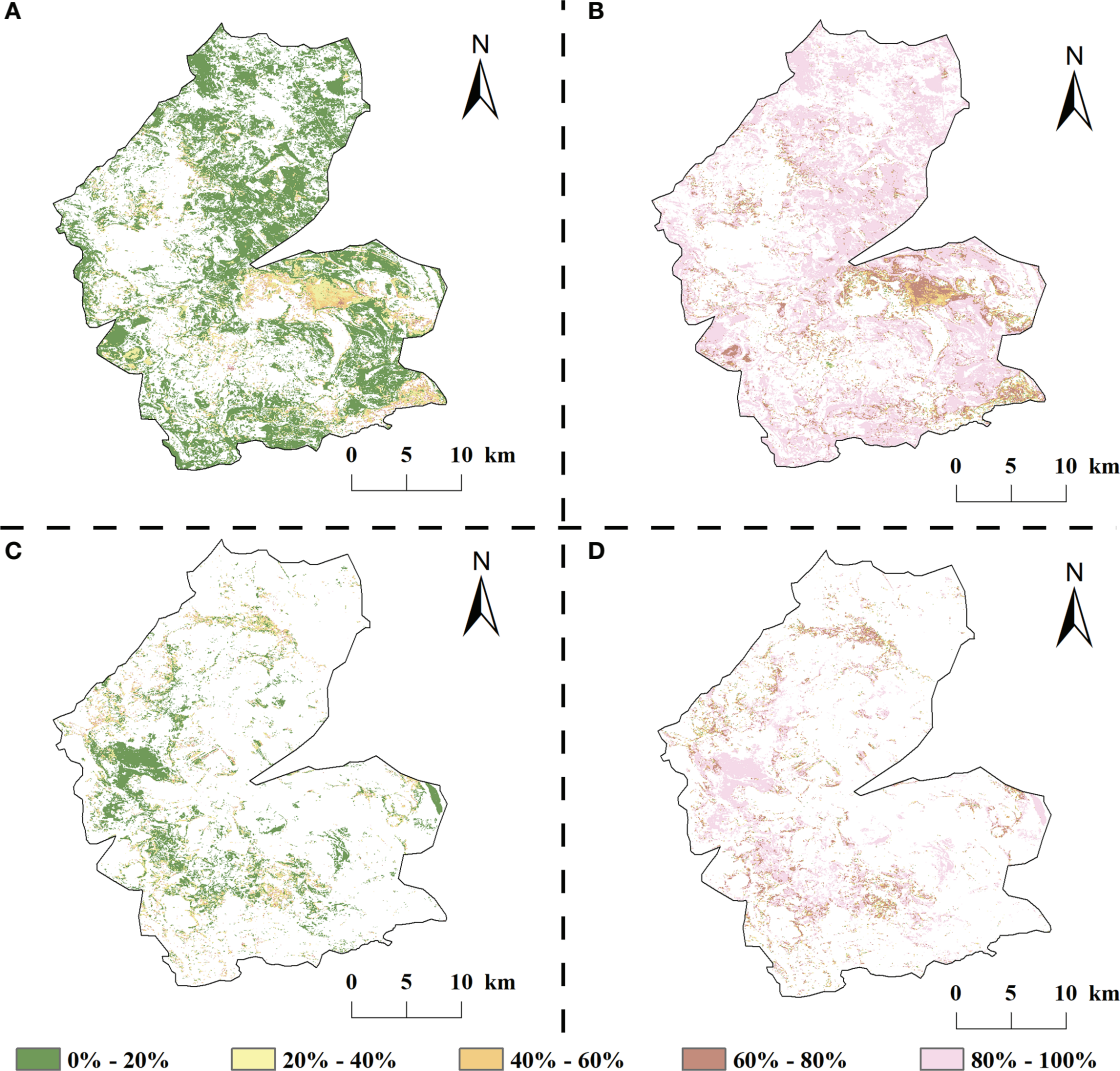
Relative contribution of climate change and human activities study areas, 1984-2020 (**A**, **B** are the relative contribution of climate change, human activities to vegetation improvement areas, respectively; **C**, **D** are the relative contribution of climate change, human activities to vegetation degradation areas, respectively).

Climate change and human activities have contributed to vegetation growth in most areas of the study area but have also resulted in a decreasing trend in local vegetation vigor (**Figure 10**). The relative contributions of climate change and human activities to vegetation improvement in the study area from 1984 to 2020 were 14.32% and 85.68%, respectively. In the vegetation improvement area of the study area, the relative contribution of climate change within 40% accounted for 91.72% of the vegetation improvement area; compared with natural factors, the contribution of human activities to vegetation improvement was mainly concentrated in the range of 80–100%, accounting for 78.20% of the vegetation improvement area and mainly distributed at the edge of the study area.

There was a large spatial variation in the magnitude of the relative contributions of climate change and climate change to vegetation degradation (**Figure 10**). In the degraded areas, although the contribution of climate change to vegetation change increased compared with the improved areas, it was still lower than the contribution of human activities (21.71% and 78.29% of the relative contribution of the two, respectively). Among them, the area where the contribution of climate change to the vegetation NDVI degradation was in the range of 0–20% respectively was the largest, accounting for 60.98% of the vegetation degradation area and mainly distributed in the western part of the study area; the area where the relative contribution of human activities was above 60% accounted for 82.64% of the vegetation degradation area and was evenly distributed in the inner part of the study area.

## 4 Discussion

### 4.1 Temporal and spatial characteristics of vegetation cover

On the time scale, the vegetation NDVI in the study area of Momoge showed a fluctuating upward trend from 1984 to 2020, and the vegetation growth status was generally good; however, the vegetation NDVI continued to decline in the middle of the study, which is consistent with the conclusion obtained by other researchers (Xue et al., 2022). In addition, the trend of high vegetation cover in the study area increased before 2000 and decreased after 2005, whereas the trend of vegetation cover and middle and high vegetation cover changed in the opposite direction. In the early stage, the study area had good vegetation cover due to abundant precipitation, sufficient heat, less human disturbance, low exploitation, and longitudinal water systems. In the middle stage, the vegetation cover decreased due to agricultural reclamation, water conservation projects, and increased extreme drought, which is consistent with the results of previous studies (Wang et al., 2022a). At the spatial scale, at least 70% of the study area showed an increasing vegetation trend from 1984 to 2020. The other decreasing areas were distributed in several villages, towns, and surrounding areas in the study area of Momo, mainly because the above areas had a poor vegetation base owing to the high intensity of human activities, and the negative human disturbance destroyed the vegetation growth environment, resulting in a decreasing vegetation cover trend.

### 4.2 Drivers and mechanisms of vegetation change

Previous studies have shown that vegetation cover change is a complex process, with long term vegetation cover change mainly influenced by natural geographic processes such as climate change, while short term vegetation change is significantly influenced by human activities such as deforestation, overgrazing and ecological engineering (Chapman et al., 2014; Qu et al., 2018). Based on the results of the driving analysis of vegetation change, we found that vegetation growth in the study area from 1984 to 2020 was driven by both climate change and human activities in 58.35% of the total study area, which shows that the joint driving factors of climate change and human activities is the main reason for the change in vegetation NDVI in the Momoge Reserve. Among them, human activities contributed more to the vegetation cover change in the study area than climate change.

#### 4.2.1 Climate change

The overall trend of vegetation versus precipitation and temperature during the growing season showed that the study area suffered from multi-year drought in the middle of the season, with precipitation below the multi-year average, temperature increased, and vegetation cover decreased; in the later season, the climate improved and vegetation cover increased as precipitation increased. The positive correlation between vegetation cover and precipitation in the study area was higher than that of temperature. The reasons for this are as follows: (1) Precipitation is a key constraint on vegetation growth and distribution in semi-arid areas (Huang and Wang, 2010). Precipitation can replenish soil moisture and promote plant growth (Shen et al., 2019). In the study area, there is severe water shortage in drought years, soil salinization is severe, water retention is poor and water content is low, and plants depend on precipitation to replenish water to maintain survival. (2) Temperature is a limiting factor affecting vegetation cover (Mao et al., 2012). In arid and semi-arid regions, increasing temperature will accelerate vegetation transpiration, resulting in increased evapotranspiration and reduced soil moisture, which will inhibit vegetation growth (Vicente-Serrano et al., 2013). With global warming, the trend of warming and drying of swampy wetlands in semi-arid areas has increased, and the frequent occurrence of drought is not conducive to local vegetation recovery.

The spatial correlation between vegetation and precipitation and temperature during the growing season showed that the NDVI response to precipitation and temperature was spatially unevenly distributed, with both positive and negative correlation coefficients. the precipitation-related areas were mostly located in meadows and croplands, where increased precipitation helped to relieve plants from water stress (Lou et al., 2015), while the temperature-related areas were mostly located in swampy wetlands or around water bubbles, where water and heat conditions were abundant and conducive to plant growth (Cai et al., 2021). The land use types in the study area are mainly swampy wetlands and croplands, unlike the more stable forest ecosystems, which are more sensitive to climate change (Piao et al., 2020; Masao et al., 2022), however, we noticed that there are fewer areas of significant correlation between vegetation cover and temperature and precipitation, and the influence of hydrothermal factors on vegetation growth activities is smaller. This is related to the implementation of ecological water recharge projects in recent years. The availability of water resources in the study area is not controlled by climatic factors but by human activities, and nowadays vegetation is rarely affected by water stress, therefore, vegetation growth is less sensitive to climatic factors, which is consistent with the results of existing studies (Shi et al., 2021).

#### 4.2.2 Human activities

In addition to climate change, human activities are also a key factor in determining the spatial distribution patterns and dynamics of vegetation in the study area, as strongly evidenced by NDVI residuals. The human land use patterns characterize the impact of human activities on the surface vegetation to a certain extent. The Momog Reserve is located in the transition zone between East Asian broadleaf forests and Eurasian steppes, where natural and artificial vegetation coexist in the reserve (Ning et al., 2014). In the study area, in the middle period, the area of swampy wetlands, woodlands and water bodies decreases with the consequent decrease in NDVI; in the later period, NDVI increases with the increase in the area of swampy wetlands, woodlands and grasslands, which is consistent with the findings of existing studies (Yang et al., 2022).

It is worth mentioning that human activities have caused a shift in land use type and use pattern in Momoge Reserve, which in turn has a positive or negative impact on vegetation cover area and quality. Irrational human cultivation and urban expansion degrade vegetation, while the implementation of ecological engineering improves it (He et al., 2021). We can see from the results of land use conversion that in the medium term, with over-exploitation by humans, a large number of vegetation NDVI high value areas (swampy wetlands, woodlands and grasslands) were converted to NDVI low value areas (water bodies, unused land and built-up land) in the study area (Mao et al., 2019); in recent years, with the implementation of ecological projects such as returning wetlands to farmland, forests to farmland, grazing to grass, and river and lake linkages (Huang et al., 2022), a large number of vegetation areas with low NDVI values (cropland and unused land) have been transformed into areas with high NDVI values (grassland and woodland) in the study area. Coupled with the gradual stabilization of cropland size in recent years, with high NDVI crops such as rice and soybean, the study area maintains a high and stable vegetation cover (Geng and Zhou, 2022). The human-driven NDVI increase in the study area is higher than the human-driven NDVI decrease in the study area, which indicates that human conservation efforts in the study area have been effective.

### 4.3 Limitations of this study

In this study, NDVI was used as a parameter to quantitatively evaluate the method of climate change and anthropogenic contribution to vegetation cover in a typical arid and semi-arid swampy region of China. However, we should also recognize the limitations of this study. First, this study used the Landsat dataset to calculate NDVI for long time series, which does not have a high spatial resolution (30 m), which may affect the study results and can be improved in the future by fusing multi-source data at a higher spatial resolution and long-term scale (Decuyper et al., 2020; Shen et al., 2022a; Shen et al., 2022b). Second, since natural factors affecting vegetation change include precipitation, temperature, topography and soil, only precipitation and temperature were considered when conducting the natural factor analysis in this paper, and multiple natural factors should be considered in the future to explore the causes of vegetation change in an integrated manner. Finally, the climate data used in this study were obtained by spatial interpolation based on sparse site data, which may be another potential source of uncertainty.

## 5 Conclusions

In this study, we found that the average NDVI of the vegetation growing season in the study area from 1984 to 2020 was 0.55, and the average annual growth rate was 0.002/a. The NDVI of 71.14% of the area increased, and there was spatial and temporal heterogeneity of vegetation cover in the study area, with a decreasing trend of low and medium vegetation cover, a generally consistent trend of medium and high vegetation cover (i.e. increasing first and then decreasing), and a decreasing trend of high vegetation cover. Spatial and temporal heterogeneity of vegetation cover in the study area was found. Among the climate change factors, precipitation is the most important factor for vegetation growth in the study area, and it is positively correlated with the NDVI in the study area as a whole and has a greater influence. Human activities also have a dual effect on vegetation change, and a series of ecological restoration measures have effectively improved the ecological environment of the area in recent years. The vegetation cover change in the study area was mainly driven by both human activities and climate change; however, the promotion in the conclusion effect of human activities on vegetation growth was greater than that of climate change, and the relative effect on the vegetation improvement area was 85.68%.

## Data availability statement

The original contributions presented in the study are included in the article/supplementary material. Further inquiries can be directed to the corresponding authors.

## Author contributions

GD: Data Analysis, writing-original draft. JG: Writing-review and editing. HJ: Project administration. DL: Formal analysis. XW and YW: Visualization. LS and CH: Investigation, Validation. All authors have read and agreed to the published version of the manuscript.

## Funding

This research was funded by the National Natural Science Foundation of China, grant numbers 41901116 and U19A2042, and the Foundation of Jilin Educational Committee, grant numbers JJKH20220448KJ.

## Acknowledgments

The authors would like to acknowledge the contributions of colleagues, institutions, and agencies that aided their efforts.

## Conflict of interest

The authors declare that the research was conducted in the absence of any commercial or financial relationships that could be construed as a potential conflict of interest.

## Publisher’s note

All claims expressed in this article are solely those of the authors and do not necessarily represent those of their affiliated organizations, or those of the publisher, the editors and the reviewers. Any product that may be evaluated in this article, or claim that may be made by its manufacturer, is not guaranteed or endorsed by the publisher.
